# Prognostic significance of KRAS gene mutations in colorectal cancer - preliminary study

**Published:** 2014

**Authors:** D Dinu, M Dobre, E Panaitescu, R Bîrlă, C Iosif, P Hoara, A Caragui, M Boeriu, S Constantinoiu, C Ardeleanu

**Affiliations:** * General and Oesophagial Surgical Department, “Carol Davila” University of Medicine and Pharmacy; “Sf. Maria” Clinical Hospital, Bucharest, Romania; ** Department of Pathology, “Victor Babes” National Institute for Research and Development in Pathology and Biomedical Sciences, Bucharest, Romania; *** Department of Biostatistics and Medical Informatics, “Carol Davila” University of Medicine and Pharmacy, Bucharest, Romania; **** Department of Pathology, “Sf. Maria” Clinical Hospital, Bucharest, Romania; ***** University of Bucharest, Faculty of Biology, Bucharest, Romania

**Keywords:** KRAS gene mutation, pyrosequencing, colorectal cancer

## Abstract

**Objective:** The prognostic significance of KRAS gene mutations, evaluated by using two methods in patients with colorectal cancer (CRC).

**Material and Methods:** Retrospective study involving 58 patients diagnosed with CRC and treated between 2003 and 2010 in the General and Esophageal Surgery Clinic of “Sf. Maria” Hospital, Bucharest. The macroscopic and microscopic examination of the resected specimens was also processed for genetic analysis in NIRDPBS, where KRAS status was determined by using two methods: PCR-RFLP and pyrosequencing.

**Results:** The clinical and biological parameters of the patients were assessed for 72 months in average. A relapse in 21 patients and a 5-year survival rate of 79.3% was discovered. The genetic analyses of KRAS gene found mutations in 22 cases (45.3%): 17 cases had mutations in codon 12, 5 cases in codon 13.

The survival rate analyses of patients with wild KRAS gene compared with the patients carrying the mutation on codon 12 /13 revealed a superposition of the survival curve.

The statistical analysis based on the TNM stage revealed different survival curves in stage I and II, shorter survival period in patients with KRAS mutation on codon 13 than in those with wild type gene (stage I - p_value=0.015; stage II - p_value=0.000).

**Conclusions:** It was not found that KRAS gene status had any prognostic significance. Nevertheless, for stage I and II patients, the mutation found on codon 13 determined a statistic significant shorter survival rate than for those with wild type. The results obtained by using the pyrosequencing method for the determination of KRAS gene status proved that it represented a reliable and reproducible method.

## Introduction

Colorectal cancer [**1**] represents a major cause of morbidity and mortality both in men and women, in the western countries. Fifty percent of the patients will die within 5 years from diagnosis, usually as a result of the metastatic disease.

Following lung cancer (for males) and breast cancer (for females), in 2008, colorectal cancer represented the second cause of cancer death in both sexes and is currently the fourth cause of death from cancer in the world [**2**].

Several genetic alterations, including chromosomal abnormalities, gene mutations, and epigenetic modifications involving several genes that regulate proliferation, differentiation, apoptosis, and angiogenesis led to the development of colorectal cancer (CRC) [**3**].

It is now widely accepted that sporadic colorectal cancers frequently arise from preneoplastic lesions through the activation of oncogenes (KRAS and BRAF) as well as the inactivation of tumor suppressor genes (APC, p16, p53, and DCC) and mismatch repair genes, such as MLH1 and MSH2 and, to a lower extent, PMS2 and hMSH6 [**4**].

KRAS is a proto-oncogene that encodes a small 21-kD guanosine triphosphate (GTP)/ guanosine diphosphate (GDP) binding the protein involved in the regulation of the cellular response to many extracellular stimuli [**5**].

KRAS is located at 12p12.1, spans approximately 38 kb, and encodes a 188–amino acid residue with a molecular weight of 21.6 kDa. KRAS normally functions in signal transduction cascades initiated by the binding of the epidermal growth factor receptor (EGFR), hepatocyte growth factor, and insulin-like growth factor to their receptors [**6**,**7**].

When activated, wild-type KRAS binds GTP, this resulting in a conformational change that allows the protein to bind and activate over 20 known downstream effectors, including Raf, Braf, mTOR, MEK1 and 2, ERK, AKT, and PIK3CA. These downstream effectors exert many different effects, including apoptosis suppression, promotion of cell growth, cell transformation, angiogenesis, migration, and differentiation [**6**,**8**].

The RAS gene family is among the most studied and best characterized of the known cancer-related genes. Of the three human ras isoforms, KRAS is the most frequently altered gene, with mutations occurring in 17%–25% of all cancers. Particularly, approximately 30%–40% of colon cancers carry a KRAS mutation. KRAS mutations in colon cancers have been associated with a poorer survival and increased tumor aggressiveness. Additionally, KRAS mutations in colorectal cancer lead to resistance to select treatment strategies.

The detection of KRAS mutations has been associated with decreased response rates to select chemotherapeutic agents. Therefore, KRAS mutational status is a critical factor when considering the use of targeted therapies. The association of KRAS gene mutation and response to therapy was first reported in patients with metastatic colorectal cancer, who were treated with anti-epidermal growth factor receptor (EGFR) agents. Lievre et al. were the first to report the link between the KRAS gene mutation and decreased response to anti-EGFR agents [**9**].

The KRAS oncogene is mutated in approximately 35%-45% of colorectal cancers, and KRAS mutational status testing has been highlighted in recent years. The most frequent mutations in this gene, point substitutions in codons 12 and 13, were validated as negative predictors of response to anti-epidermal growth factor receptor antibodies. Therefore, determining the KRAS mutational status of tumor samples has become an essential tool in managing patients with colorectal cancers.

In this study, the prevalence and distribution of KRAS mutations and correlation with overall survival were assessed.

## Material and Methods

The study group comprised 58 patients diagnosed with colorectal cancer, treated and followed up during 2003-2010 in the General and Esophageal Surgery Clinic of “Sf. Maria” Hospital. The analysis was performed in a retrospective manner. Patients were diagnosed by using imaging and endoscopic biopsy and 57 were surgically treated by resection with a radical intent. The macroscopic analyses of the surgical specimens observed the tumor location, degree of parietal invasion, the involvement of the resection margins and the identification of loco-regional lymph nodes.

The microscopic examination was performed in the Laboratory of Pathology of “Sf. Maria” Hospital, by assessing: histological type, grading, mucus production, vascular and perineural invasion, degree of parietal invasion (pT), microscopic invasion of the resection margins, metastases in lymph nodes.

All the patients were TNM staged, with a complementary treatment according to the disease stage. Patients were periodically followed up by clinical examination, tumor markers (CA19-9, CEA), colonoscopy, CT. After the standard histopathological examination, the biological samples were analyzed by specific methods in order to highlight the KRAS gene mutations in NIRDPBS Bucharest.

**Tissue samples**

The study included 58 tumour samples from patients with CRC. Serial 5–10 sections (5 µm) from the buffered formalin fixed, paraffin embedded (FFPE) tissue were cut from each paraffin block. The first section was stained with H&E and the histopathological diagnosis was performed, making sure that >80% of the test area was represented by tumoral tissue.

**DNA extraction**

All the procedures were taken to prevent the DNA contamination. Genomic DNA was isolated according to the manufacturer’s protocols with QIAamp DNA Mini Kit (Qiagen, Hilden, Germany). All the tumor sections were placed in two baths of xylene for deparaffinization and after, for rehydratation, in two baths of ethanol. For a complete lysis, they were placed in the lysis buffer with proteinase K at 56°C overnight. After lysis, DNA was precipitated with ethanol and was fixated on the QIAamp silica membrane by centrifugation. Two washes were performed and the purified DNA was eluted in buffer AE or water. The DNA concentration and purity were determined spectrophotometrically at 230, 260 and 280 nm by using the NanoDropACTGene ASP-3700 USA. Mutations in KRAS gene were detected by pyrosequencing (codons 12, 13, 61) and by PCR-RFLP (codons 12 and 13).

**Pyrosequencing**

Pyrosequencing analysis was performed according to the manufacturer’s protocols (Therascreen KRAS Pyro Kit Handbook, version 1, July 2011) by using CE-IVD marked PyroMark KRAS kit (QIAGEN, Hilden, Germany). For each sample, 10 ng of genomic DNA were amplified for the analysis of mutations in codons 12 and 13 and another 10 ng DNA for mutations in codon 61.

Each PCR product was analysed by pyrosequencing by using PyroMark Gold Q96 reagents, Streptavidin Sepharose High Performance (GE Healthcare Bio-Science AB, Uppsala, Sweden), in a PyroMark Q24 instrument (QIAGEN, Hilden, Germany) and PyroMark Q24 1.0.6.3 software.

**PCR-RFLP**

For codon 12, the PCR reactions contained 1X reaction buffer, 1,5 mM magnesium chloride, 1,5 units of PlatinumTaq DNA polymerase (Invitrogen, Brasil), 0,2 mMdeoxynucleotide triphosphates (dATP, dGTP, dCTP, dTTP), 1 mM of each primer, and 500 ng of genomic DNA in 25 µL total volume. The primers sequences were the following [**10**]:

(Sense) ACTGAATATAAACTTGTGGTAGTTGGACCT,

(Antisense) TAATATGTCGACAAAACAAGATTTACCTC

After the amplification, fragments of 135 base pairs were incubated with Mva I (BstNI) (Fermentas) for restriction. MvaI determined the cleavage of the wild type fragment in codon 12 in two fragments with sizes of 106 and 29 base pairs, since the mutation presence in codon 12 would cancel the restriction situs for MvaI. A mutant case has both alleles, normal and mutant.

PCR was performed in a thermocycler (Gene Amp PCR System 2f00, Applied Byosistems, Singapore), it comprised 45 cycles and each cycle was performed for denaturation at 940C for 1 min, for annealing at 550C for 1 min and 720C for 30 s for extension.

Electrophoresis was performed by using 2% agarose gel. The electrophoresis gel was stained with ethidium bromide and photographed by transilluminator (Biolmaging Systems Digi Doc - It System, Upland, USA).

For codon 13, the PCR reactions contained the same components except for the primers sequences and concentration (1,25mM) and the annealing temperature (500C). The sequences for primers for codon 13 were the following [**11**]:

(Sense)5′-GTACTGGTGGAGTATTTGATAGTGTATTAA-3′

(Antisense) 5′-GTATCGTCAAGGCACTCTTGCCTAGG-3′.

After amplification, 12,5µL PCR products (159 base pairs) were incubated with HaeIII (Fermentas). The wild-type allele was cleaved in three fragments of 85, 48 and 26 pair base while the mutant allele was cleaved in only two fragments of 85 and 74 bp. Electrophoresis was performed by using 4% HR agarose gel.

**Quality control**

Two DNA samples with known mutational status for KRAS gene codons 12 and 13 were used for each PCR-RFLP test: wild type DNA (negative control - NC), mutant DNA (positive control - PC) and no template control (NTC). For pyrosequencing, a sample with unmethylated control DNA was included, provided by the kit as a positive control for PCR, and sequencing reactions and a NTC in every run.

## Results

In our study, 13 patients had the primary tumor located on the right colon, 28 patients on the left colon and 17 on the rectum. 42 patients presented the common type adenocarcinoma and 16 developed mucus-secreting adenocarcinoma. The following grading types were assessed: G1 - 3 patients, G2 - 51 patients and G3 - 4 patients. TNM staging was the following: stage I -12 patients, stage II - 17 patients, stage III - 23 patients and stage IV - 6 patients. 56 patients underwent adjuvant therapy, consisting in chemotherapy (5-FU in 40 patients, 14 patients and PCT FOLFOX + Bevazucimab in 2 patients) or radiotherapy - 8 patients.

Postoperative follow-up consisted in clinical, imaging and laboratory monitoring for a mean period of 64 months (6 months to 144 months). 27 patients developed recurrence as following–loco-regional - 11 patients, distant metastases – 13 patients and 3 patients developed both local recurrence and distant metastases, leading to the continuation of an appropriate therapy. The 3-year survival rate was 89.2%, whereas the 5-year survival rate was 75.6%. The follow-up ended on 31.12.2013 and the survival rate at the end of the follow-up was 72.5%.

The samples were analyzed for mutations in codons 12 and 13 by using both methods, in a blind manner. All cases were analyzed by using both methods (PCR-RFLP or pyrosequencing) for KRAS mutations, identifying 22 cases with this mutation (**Table 1**): 17 cases presented codon 12 mutations, 5 cases presented codon 13 mutations.

**Table 1:**
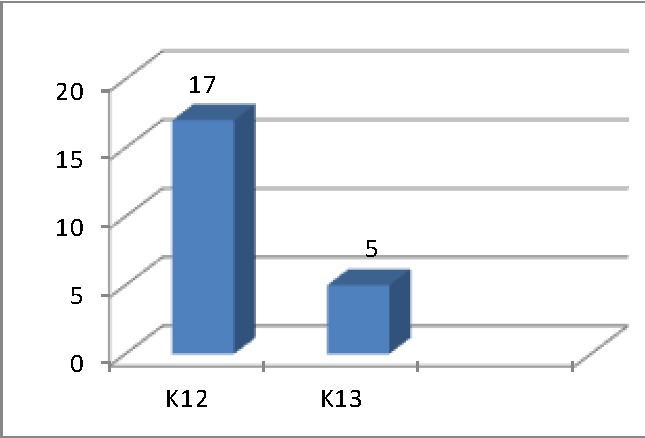
The distribution of KRAS gene mutation in codons 12 and 13

**PCR-RFLP results**

**Fig. 1:**
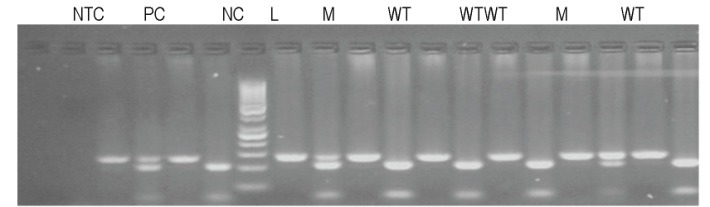
Gel electrophoresis, agarose 2% (w/v) for codon 12. PCR product (left, band at 135 bp) and restriction fragments (right) were migrated for each sample; NTC – no template control; PC – positive control (mutant heterozygous, bands at 135, 106 and 29 bp); NC – negative control (wild type, bands at 106 and 29 bp); WT – wild type sample, M – mutant sample; L – DNA molecular weight markers (GeneRuler 50 pb DNA Ladder, Fermentas)

The image for a wild type sample for KRAS, codon 12 mutation showed two bands of 106 base pairs (bp) and 29 bp, while a mutant case showed three bands: one of 135 bp (mutant allele) and two of 106 bp, 29 bp (normal allele) (**Fig. 1**).

**Fig. 2:**
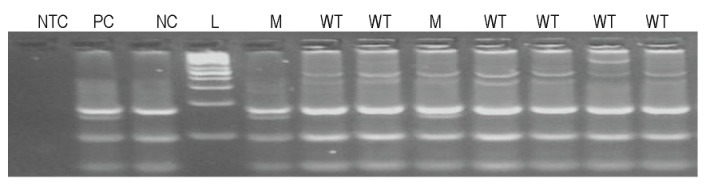
Gel electrophoresis, agarose HR 4% (w/v) for codon 13, of restriction fragments. NTC – no template control; PC – positive control (mutant heterozygous, bands at 85, 74, 48 and 26 bp); NC – negative control (wild type, bands at 85, 48 and 26 bp); WT – wild type sample, M – mutant sample; L – DNA molecular weight markers (GeneRuler 50 pb DNA Ladder, Fermentas)

The image for a wild type sample showed three fragments of 85, 48 and 26 pb, while a mutant case four bands of 85 and 74 bp (mutant allele) and 85, 48 and 26 bp (normal allele) – (**Fig. 2**).

**Pyrosequencing results**

**Fig. 3:**
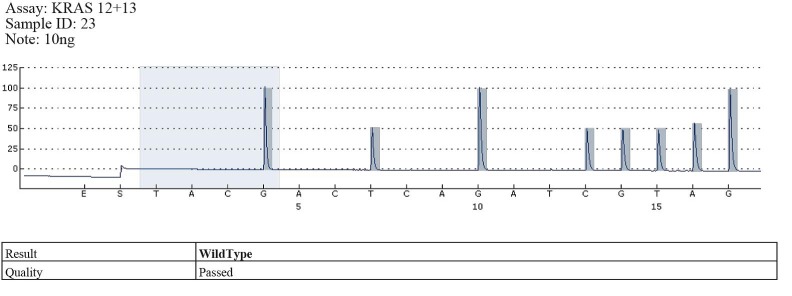
Example of wild type - codons 12, 13

Codon 12 mutations were identified in 17 cases and wild type in the rest of them (**Fig. 3**).

**Fig. 4:**
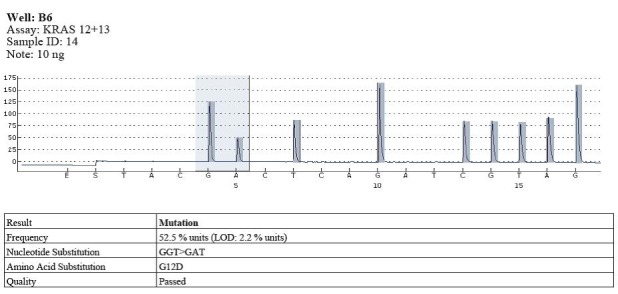
Example of mutation in codon 12 (GGT>GAT)

**Fig. 5:**
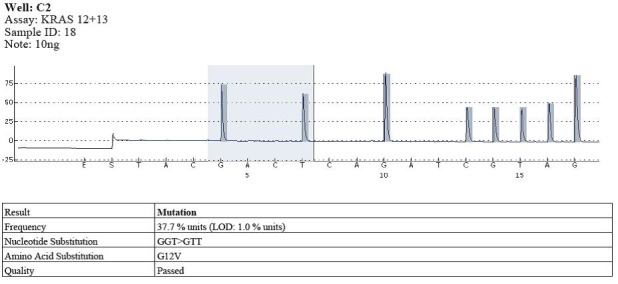
Example of mutation in codon 12 (GGT>GTT)

**Fig. 6:**
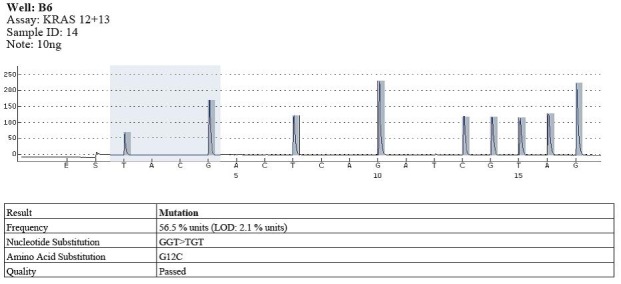
Example of mutation in codon 12 (GGT>TGT)

The types of substitutions were identified in 17 out of the 58 cases of pyrosequencing patients analyzed with KRAS gene mutations in codon 12: 11 cases with substitution type GGT> GAT, the substituted amino acid being G12D; 5 cases with substitution type GGT> GTT, the substituted amino acid being G12V; 1 case with substitution type GGT> TGT, the substituted amino acid being G12C (**Fig. 4**-**6**).

**Fig. 7:**
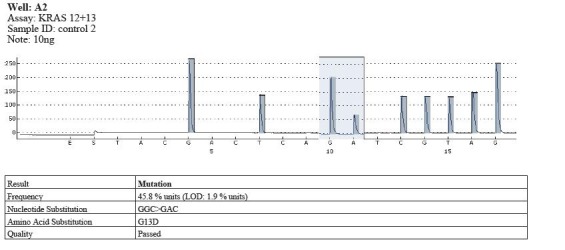
Example of mutation in codon 13 (GGC>GAC)

Mutations in codon 13 were identified in 5 cases, and wild type in the rest. The mutation type was GGC> GAC and the substituted amino was G13D (**Fig. 7**).

**Fig. 8:**
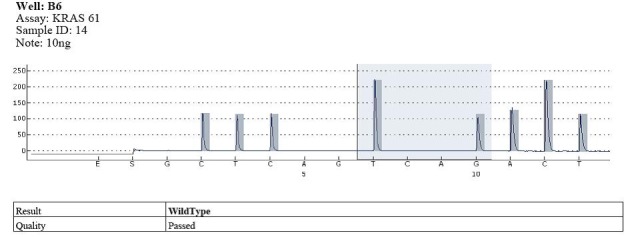
Example of wild type – codon 61

All the cases were studied by pyrosequencing were wild type in codon 61 (**Fig. 8**).

**Statistical analysis**

The survival analysis did not show significant differences in survival between patients with wild gene and the KRAS mutation.

**Statistical analysis of survival in TNM stages:**

**Mutationin codon K13:**

The analysis of the patient’s survival within each TNM stage, looking as prognostic factor mutation at codon 13, showed that there are differences in survival in patients in stage I and II, with a reduced survival rate in patients with this mutation in codon 13.

**Stage_TNM=I**

**Table 2:** Test of equality of survival distributions for the different levels of Codon_K13 in stage I

	Case Processing Summary a			
		N of Events	Censored **(Survivors)**	
Codon_K13	Total N	**Deceased**	N	Percent
yes	3	2	1	33.3%
no	8	0	8	100.0%
Overall	11	2	9	81.8%
a. Stage_TNM_Simpl = I				
				
				
	Overall Comparisons a		
	Chi-Square	df	Sig. **(p_value)**
Log Rank (Mantel-Cox)	5.926	1	**0.015**
Breslow (Generalized Wilcoxon)	5.926	1	**(0.015**
Test of equality of survival distributions for the different levels of Codon_K13.			
a. Stage_TNM_Simpl = I			

Out of the 11 patients in TNM stage I, 8 patients with wild type KRAS gene had a 100% survival rate. Mutations in codon 13 resulted in a different survival curve than in patients with the wild type; so this mutation in codon 13 in TNM stage I patients may be considered a bad prognostic factor (Test: Log Rank, **p_value = 0.015**) (**Table 2**).

**Stage_TNM=II**

**Table 3:** Test of equality of survival distributions for the different levels of Codon_K13 in stage II

	Case Processing Summary a			
		N of Events	Censored **(Survivors)**	
Codon_K13	Total N	**Deceased**	N	Percent
yes	1	1	0	0.0%
no	16	2	14	87.5%
Overall	17	3	14	82.4%
a. Stage_TNM_Simpl = II				
				
				
	Overall Comparisons a		
	Chi-Square	df	Sig. **(p_value)**
Log Rank (Mantel-Cox)	16.000	1	**0.000**
Breslow (Generalized Wilcoxon)	16.000	1	**0.000**
Test of equality of survival distributions for the different levels of Codon_K13.			
a. Stage_TNM_Simpl = II			

Of the 17 patients in TNM stage II, one patient had mutation in codon 13 and died, resulting in a survival rate of 0%, leading to statistically significant survival differences. KRAS mutations in codon 13 could be considered poor prognostic factor in patients in TNM stage II (Test: Log Rank, **p_value = 0.000**) (**Table 3**).

**Stage III (23 patients)**

In the 23 patients in TNM stage III, according to KRAS gene variants, no statistically significant differences between the survival curves or occurrence of relapse were found.

**Stage IV (6 patients)**

6 patients were in TNM stage IV - 3 patients with KRAS wild-type gene (2 with peritoneal carcinomatosis and one patient with bone metastases) and 3 patients with KRAS mutations in codon 12 (2 with liver metastases and one with single brain metastasis). There was a statistical significant difference in survival between patients with mutations in the KRAS gene and patients with wild type gene, showing a better survival in patients with mutant type (**p_value = 0.025**). The explanation could be the differences in the therapeutic approach: patients with liver metastases received targeted therapy with VEGF antibody; in patients with single brain metastases metastasectomy with favorable outcome was performed; patients with peritoneal carcinomatosis or bone metastases received less efficient therapeutic options.

## Discussion

The genetic and epigenetic changes may affect the survival of the patients with colorectal cancer. In order to analyze the survival rate of the patients with colorectal cancer we only focused on the status of KRAS mutation. Our study was a retrospective analysis performed on a series of cases from one single institution (“Sf. Maria” Clinical Hospital). The follow-up of patients was performed during a period of time between 6 and 144 months and it was only possible on a small size group.

The frequency of KRAS gene mutations was of 40%, which is comparable with the results presented by the other studies in literature, that showed that approximately 40-65% of colorectal cancers present KRAS mutation [**12**,**13**] in codons 12 and 13 in exon 2 in particular, and less frequently in 61, 64 [**14**,**15**].

Data collected from literature showed that the KRAS mutation is mainly present in codon 12 and 13, meaning 95% of all mutations: 80% in codon 12, 15% in codon 13; other mutations in codons 61, 146, 154 rarely appear, representing about 5% of all the mutations. The most frequent mutation in codon 12 was G12V and G12D, and in codon 13 - G13D [**16**].

In our study group, we sought mutations in codons 12, 13, 61. Mutations in codon 12 were accounted for 77.2% of the mutations; the remaining being in codon 13; using pyrosequencing mutations they were identified in codon 61. Similar results were found in literature when talking about the types of substitutions: in codon 12 - G12D in 11/17 cases, G12V in 5/17 cases and G12C 1/17 cases; we have found G13D mutation in codon 13 in 5/5 cases.

Different biological responses may be influenced by genetic mutations in different codons. Thus, while the mutation of codon 12 was observed to be associated with mucinous colorectal carcinoma, codon 13 mutation characterizes the non-mucinous cancers, but with increased aggressiveness and increased metastatic potential and also occur with increased frequency in metastatic lymph nodes [**17**,**18**].

Different results were found in the study group compared to the ones in literature: among 16 mucus-secreting adenocarcinomas, 8 cases presented wild type KRAS gene and 6 cases presented codon 12 mutations and 2 cases presented codon 13 mutations. The other 11 cases with codon 12 mutations were common type adenocarcinomas; 2 of the 5 patients with codon 13 mutation had mucinous tumors.

The data obtained was different than data in other studies: in terms of aggressiveness - 4 patients presented type G3 and only one patient presented a mutation in codon 13 and 2 patients presented mutations in codon 12; out of the 26 patients, who showed lymph node metastases, 7 had mutation in codon 12 and only one had a mutation in codon 13.

When talking about the different treatment strategies in CRC, KRAS gene mutation has both a prognostic and predictive value, being the most studied and promising biomarker.

Most studies showed that the KRAS gene mutations may be a poor prognostic factor as opposed to the results of our study where there was no confirmed prognosis value of these mutations; the 22 mutations in KRAS gene did not cause statistically significant differences in survival compared to wild-type KRAS gene [**19**].

The survival analysis of the study group showed that KRAS mutation in codon 13 might be considered a poor prognostic factor for patients in stage I and II, while the mutation in codon 12 did not influence overall survival.

Several clinical trials examined the prognostic role: RASCAL study conducted on 2721 patients from 13 countries showed that the presence of KRAS mutation increased the risk of relapse and death. PETACC3 translational study showed that the KRAS mutation had no major prognostic value in patients with CRC TNM stages II and III, the same as in our study [**20**].

Many studies stated that stage III patients who present KRAS mutations display a significantly worse disease-free survival period than those with wild-type KRAS [**21**,**22**], which might be partially explained by the impact of either KRAS12 or KRAS13 mutations on prognosis, but the results are different from our study.

In patients with I and II TNM stages, in the study group, the KRAS mutation in codon 13 was correlated with a statistically significant lower survival rate and there was no proof that the mutation in codon 12 would have a prognostic role. There are no studies in literature regarding the prognostic role of KRAS mutations in TNM stage I and II colorectal cancer.

Implementing gene testing involves choosing the optimal method for the determination of the KRAS gene status. Currently, there is no standard protocol in the mutation testing; there are significant differences between the frequency of KRAS gene mutation in patients with CRC from different continents: Asia - 24%, Europe - 36% and Latin America - 40%. Although it is less frequently found in Asia, China yet reported a 33% frequency of KRAS mutation in CRC, mutation determined by using nucleotide-sequencing DNA. The differences in the mutational status of KRAS gene may be explained by the following: processed tissue type, the percentage of cancerous cells in the analyzed sample, the quality of the extracted DNA, the test method and the objective of the test.

The nowadays trend is the use of personalized treatment that means the application of tailored therapeutic protocols, including minimally invasive surgery combined with PCT and monoclonal antibodies.

The identification of KRAS gene mutation and testing the types of mutation confers a new opening for the personalized medicine. Applying an individualized treatment, not only benefits the patient by reducing the side effects and improving survival, but also the health care system by lowering costs.

## Conclusions

In our study, we did not find that the KRAS gene status has any prognostic significance; patients presented a superposition of the survival curves. Nevertheless, for stage I and II patients, the mutation found on codon 13 determines a statistic significant shorter survival rate than those with wild type. The results obtained by using the pyrosequencing method for the determination of KRAS gene status proved that it represents a reliable and reproducible method.

**Acknowledgments**

This work was supported by the strategic grant POSDRU/159/1.5/S/133391 and POSCCE 173/2010.
